# How splicing confers treatment resistance in prostate cancer

**DOI:** 10.7554/eLife.82070

**Published:** 2022-08-23

**Authors:** Prathyusha Konda, Srinivas R Viswanathan

**Affiliations:** 1 Dana-Farber Cancer Institute, Harvard Medical School Boston United States

**Keywords:** AR-V7, prostate cancer, nuclear import, intranuclear mobility, transcriptional activity, androgen receptor, Human

## Abstract

A splice variant of the androgen receptor that drives prostate cancer resistance translocates into the nucleus using a different mechanism from the full-length receptor and exhibits distinct molecular properties once inside.

**Related research article** Kim S, Au CC, Jamalruddin MAB, Abou-Ghali NE, Mukhtar E, Portella L, Berger A, Worroll D, Vatsa P, Rickman DS, Nanus DM, Giannakakou P. 2022. AR-V7 exhibits non-canonical mechanisms of nuclear import and chromatin engagement in castrate-resistant prostate cancer. *eLife* **11**:e73396. doi: 10.7554/eLife.73396.

Prostate cancer is the second leading cause of cancer-related deaths among men and claims over 350,000 lives worldwide each year. Most prostate cancers are driven by male hormones called androgens, which bind to and activate the androgen receptor (AR). Once an androgenic ligand, such as testosterone, binds to the androgen receptor, the receptor-ligand complex travels from the cytoplasm of the cell into its nucleus. Within the nucleus, the activated androgen receptor works as a transcription factor, which binds to DNA and triggers the cell to transcribe genes that are involved in prostate cancer progression. Therefore, androgen deprivation therapy, which lowers the levels of androgens in the body through drugs or surgical castration, represents an effective frontline treatment for prostate cancer.

While most prostate cancers initially shrink following treatment, resistance invariably emerges and the cancer relapses. This leads to the development of castration-resistant prostate cancer (CRPC), which can grow and spread to different parts of the body despite low levels of androgens. Multiple studies have shown that one of the most common molecular alterations seen in CRPC is the re-activation of signaling through the androgen receptor. This has led to the development of androgen receptor pathway inhibitors (ARPIs) that either block the part of the AR that binds to the ligand (thus preventing the receptor from becoming active) or interfere with the synthesis of androgens (Chen et al., 2004; Robinson et al., 2015; Visakorpi et al., 1995; Watson et al., 2015).

Unfortunately, many CRPCs develop resistance even to these drugs. It is thought that one of the mechanisms through which this resistance develops may be the production of splice variants of the androgen receptor. A splice variant is a version of a protein that results from ‘alternative splicing’ of the mRNA before it is translated into a protein. When an mRNA molecule is transcribed from the genome, it is spliced to remove sequences that do not code for the protein (known as introns), and to join together the remaining coding regions (known as exons). If this splicing process is modified, for example, due to a mutation, this can lead to different regions being spliced in or out of the mRNA. Consequently, different versions of the protein, or splice variants, are translated. In the case of the androgen receptor, splice variants that preserve the DNA-binding domain of the protein but lack the ligand-binding domain allow the protein to continue driving androgen receptor signaling even under low-androgen conditions (Figure 1; Dehm et al., 2008; Watson et al., 2015; Westaby et al., 2022).

**Figure 1.**
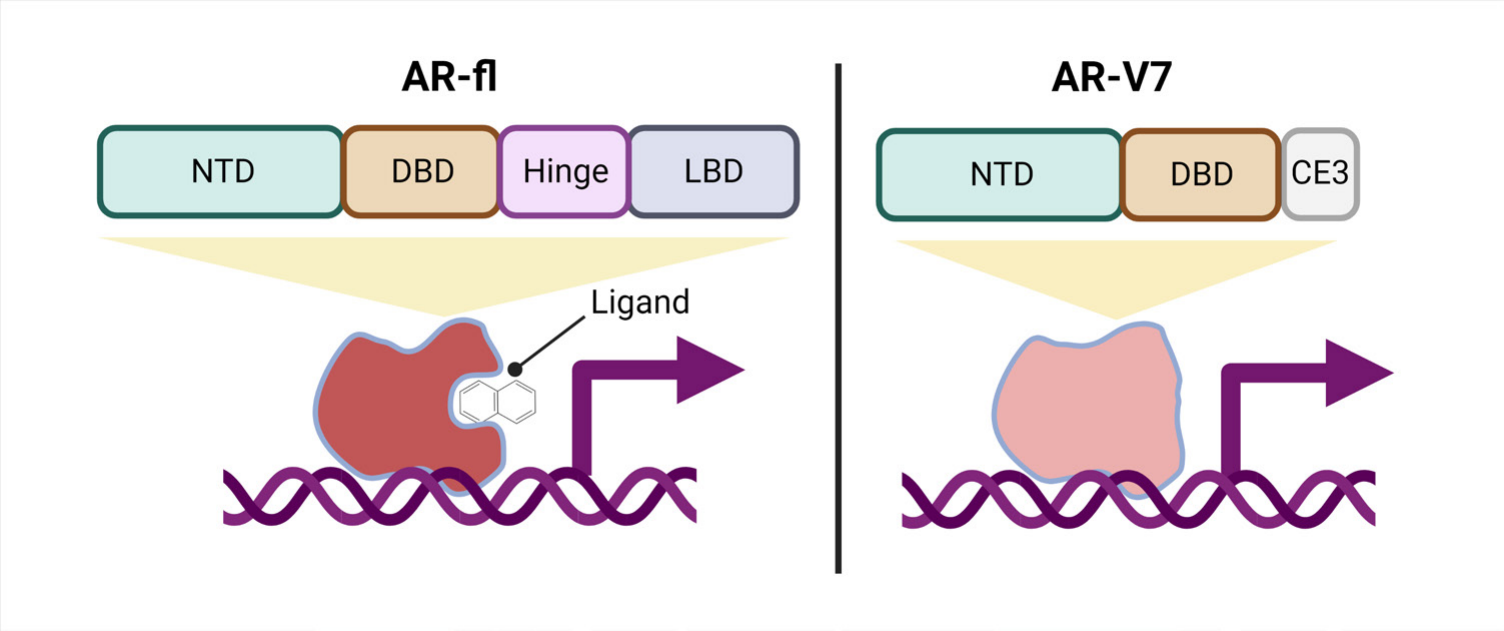
Schematic of a full-length androgen receptor (AR) compared to splice variant AR-V7. The full-length androgen receptor (AR-fl, left) binds to androgenic ligands via its ligand binding domain (LBD; dark grey rectangle) and activates the transcription of downstream genes. This form of the protein has a hinge domain (purple rectangle), which is required for the receptor to translocate into the nucleus, as well as a DNA binding domain (DBD; orange rectangle) and an N-terminal domain (NTD, green rectangle). Comparatively, a truncated splice variant of the receptor, called AR-V7 (right), only contains an NTD and a DBD followed by a cryptic exon (CE3; light grey rectangle), and lacks a hinge domain and an LBD. This version of the androgen receptor protein can activate downstream genes without binding to a ligand.

One important mechanism of resistance to ARPIs is the production of different androgen receptor splice variants. The most well-studied, AR-V7, is a truncated receptor that results when exons four to eight of the full-length mRNA sequence are missing. AR-V7 lacks the ligand binding domain of the full-length version, allowing it to signal in the absence of an androgenic ligand (Figure 1). The truncated receptor is both a biomarker of CRPC and a possible contributor to ARPI resistance (Antonarakis et al., 2014; Armstrong et al., 2019; Luo et al., 2018; Sharp et al., 2019; Westaby et al., 2022).

Interestingly, AR-V7 also lacks the hinge domain that the full-length receptor requires to translocate from the cytoplasm to the nucleus (Figure 1; Thadani-Mulero et al., 2014), leading to the question of how AR-V7 moves into the nucleus and exerts its transcriptional activity. Now, in eLife, Paraskevi Giannakakou and colleagues from Weill Cornell Medical College – including Seaho Kim, CheukMan Cherie Au, and Mohd Azrin Bin Jamalruddin as joint first authors – report how AR-V7 enters the nucleus (Kim et al., 2022).

Using microscopy to image live cells over time, Kim et al. demonstrated that, similar to the full-length receptor, AR-V7 is imported into the nucleus relatively quickly. However, AR-V7 does not rely on microtubules or importin – two protein complexes involved in the nuclear transport of the full-length version. Additionally, Kim et al. implicate the zinc finger (D-box) domain of AR-V7 in the nuclear import of the truncated receptor (which is not the case for the full-length receptor).

Fluorescence recovery after photobleaching, combined with other advanced microscopy techniques, demonstrated that AR-V7 is constantly moving within the nucleus, and does not stay in contact with the same region of DNA for long periods of time. This contrasts with the full-length counterpart (and other nuclear hormone receptors), which stay on the same region of DNA for prolonged durations, and as such, exhibit comparatively less movement within the nucleus.

These findings point to a ‘hit-and-run’ transcription model for AR-V7, in which it transiently binds to DNA sequences and recruits secondary transcription factors that keep the target gene active, even after it unbinds. While hit-and-run transcription has been typically associated with proteins that repress transcription, it has been proposed that AR-V7 may have repressive activity in CRPC (Cato et al., 2019). Intriguingly, Kim et al. found that AR-V7 also promotes the nuclear translocation of full-length androgen receptor without its ligand, although the exact mechanism remains unclear. It is also unknown how this may impact AR signaling. This information may be clinically relevant, as it is common for patients with CRPC to co-express the full-length receptor and spliced variant AR-V7 (Watson et al., 2010).

Taken together, these findings shed light on important distinctions between spliced AR-V7 and full-length androgen receptor, although several open questions remain. Further studies will be needed to identify which proteins transport AR-V7 into the nucleus. Additionally, it will be important to determine the link between the hit-and-run activity of AR-V7 and the function of this spliced receptor. Finally, the extent to which the mechanisms elucidated by Kim et al. apply to other spliced variants of the androgen receptor merits further investigation. More broadly, a thorough understanding of how the full-length androgen receptor is mechanistically distinct from its splice variants may provide opportunities to selectively block the variants from signaling in advanced prostate cancer.

## References

[bib1] Antonarakis ES, Lu C, Wang H, Luber B, Nakazawa M, Roeser JC, Chen Y, Mohammad TA, Chen Y, Fedor HL, Lotan TL, Zheng Q, De Marzo AM, Isaacs JT, Isaacs WB, Nadal R, Paller CJ, Denmeade SR, Carducci MA, Eisenberger MA, Luo J (2014). AR-V7 and resistance to enzalutamide and abiraterone in prostate cancer. The New England Journal of Medicine.

[bib2] Armstrong AJ, Halabi S, Luo J, Nanus DM, Giannakakou P, Szmulewitz RZ, Danila DC, Healy P, Anand M, Rothwell CJ, Rasmussen J, Thornburg B, Berry WR, Wilder RS, Lu C, Chen Y, Silberstein JL, Kemeny G, Galletti G, Somarelli JA, Gupta S, Gregory SG, Scher HI, Dittamore R, Tagawa ST, Antonarakis ES, George DJ (2019). Prospective multicenter validation of androgen receptor splice variant 7 and hormone therapy resistance in high-risk castration-resistant prostate cancer: the PROPHECY study. Journal of Clinical Oncology.

[bib3] Cato L, de Tribolet-Hardy J, Lee I, Rottenberg JT, Coleman I, Melchers D, Houtman R, Xiao T, Li W, Uo T, Sun S, Kuznik NC, Göppert B, Ozgun F, van Royen ME, Houtsmuller AB, Vadhi R, Rao PK, Li L, Balk SP, Den RB, Trock BJ, Karnes RJ, Jenkins RB, Klein EA, Davicioni E, Gruhl FJ, Long HW, Liu XS, Cato ACB, Lack NA, Nelson PS, Plymate SR, Groner AC, Brown M (2019). ARv7 represses tumor-suppressor genes in castration-resistant prostate cancer. Cancer Cell.

[bib4] Chen CD, Welsbie DS, Tran C, Baek SH, Chen R, Vessella R, Rosenfeld MG, Sawyers CL (2004). Molecular determinants of resistance to antiandrogen therapy. Nature Medicine.

[bib5] Dehm SM, Schmidt LJ, Heemers HV, Vessella RL, Tindall DJ (2008). Splicing of a novel androgen receptor exon generates a constitutively active androgen receptor that mediates prostate cancer therapy resistance. Cancer Research.

[bib6] Kim S, Au CC, Jamalruddin MAB, Abou-Ghali NE, Mukhtar E, Portella L, Berger A, Worroll D, Vatsa P, Rickman DS, Nanus DM, Giannakakou P (2022). AR-V7 exhibits non-canonical mechanisms of nuclear import and chromatin engagement in castrate-resistant prostate cancer. eLife.

[bib7] Luo J, Attard G, Balk SP, Bevan C, Burnstein K, Cato L, Cherkasov A, De Bono JS, Dong Y, Gao AC, Gleave M, Heemers H, Kanayama M, Kittler R, Lang JM, Lee RJ, Logothetis CJ, Matusik R, Plymate S, Sawyers CL, Selth LA, Soule H, Tilley W, Weigel NL, Zoubeidi A, Dehm SM, Raj GV (2018). Role of androgen receptor variants in prostate cancer: report from the 2017 Mission Androgen Receptor Variants meeting. European Urology.

[bib8] Robinson D, Van Allen EM, Wu YM, Schultz N, Lonigro RJ, Mosquera JM, Montgomery B, Taplin ME, Pritchard CC, Attard G, Beltran H, Abida W, Bradley RK, Vinson J, Cao X, Vats P, Kunju LP, Hussain M, Feng FY, Tomlins SA, Cooney KA, Smith DC, Brennan C, Siddiqui J, Mehra R, Chen Y, Rathkopf DE, Morris MJ, Solomon SB, Durack JC, Reuter VE, Gopalan A, Gao J, Loda M, Lis RT, Bowden M, Balk SP, Gaviola G, Sougnez C, Gupta M, Yu EY, Mostaghel EA, Cheng HH, Mulcahy H, True LD, Plymate SR, Dvinge H, Ferraldeschi R, Flohr P, Miranda S, Zafeiriou Z, Tunariu N, Mateo J, Perez-Lopez R, Demichelis F, Robinson BD, Sboner A, Schiffman M, Nanus DM, Tagawa ST, Sigaras A, Eng KW, Elemento O, Sboner A, Heath EI, Scher HI, Pienta KJ, Kantoff P, de Bono JS, Rubin MA, Nelson PS, Garraway LA, Sawyers CL, Chinnaiyan AM (2015). Integrative clinical genomics of advanced prostate cancer. Cell.

[bib9] Sharp A, Coleman I, Yuan W, Sprenger C, Dolling D, Rodrigues DN, Russo JW, Figueiredo I, Bertan C, Seed G, Riisnaes R, Uo T, Neeb A, Welti J, Morrissey C, Carreira S, Luo J, Nelson PS, Balk SP, True LD, de Bono JS, Plymate SR (2019). Androgen receptor splice variant-7 expression emerges with castration resistance in prostate cancer. The Journal of Clinical Investigation.

[bib10] Thadani-Mulero M, Portella L, Sun S, Sung M, Matov A, Vessella RL, Corey E, Nanus DM, Plymate SR, Giannakakou P (2014). Androgen receptor splice variants determine taxane sensitivity in prostate cancer. Cancer Research.

[bib11] Visakorpi T, Hyytinen E, Koivisto P, Tanner M, Keinänen R, Palmberg C, Palotie A, Tammela T, Isola J, Kallioniemi OP (1995). In vivo amplification of the androgen receptor gene and progression of human prostate cancer. Nature Genetics.

[bib12] Watson PA, Chen YF, Balbas MD, Wongvipat J, Socci ND, Viale A, Kim K, Sawyers CL (2010). Constitutively active androgen receptor splice variants expressed in castration-resistant prostate cancer require full-length androgen receptor. PNAS.

[bib13] Watson PA, Arora VK, Sawyers CL (2015). Emerging mechanisms of resistance to androgen receptor inhibitors in prostate cancer. Nature Reviews Cancer.

[bib14] Westaby D, Fenor de La Maza M, Paschalis A, Jimenez-Vacas JM, Welti J, de Bono J, Sharp A (2022). A new old target: androgen receptor signaling and advanced prostate cancer. Annual Review of Pharmacology and Toxicology.

